# In Vitro Genotoxicity Assessment of Commercially Available Graphene Quantum Dots in Human Peripheral Blood Cells and Salivary Leukocytes

**DOI:** 10.3390/toxics14060523

**Published:** 2026-06-15

**Authors:** Tamara Ćetković Pećar, Irma Durmišević, Mirta Milić, Anja Haverić, Maida Hadžić Omanović, Sanjin Gutić, Bojana Žegura, Sanin Haverić

**Affiliations:** 1 Institute for Genetic Engineering and Biotechnology, University of Sarajevo, Zmaja od Bosne 8, 71000 Sarajevo, Bosnia and Herzegovina; tamara.cetkovic@ingeb.unsa.ba (T.Ć.P.); irma.durmisevic@ingeb.unsa.ba (I.D.); anja.haveric@ingeb.unsa.ba (A.H.); maida.hadzic@ingeb.unsa.ba (M.H.O.); 2 Division of Toxicology, Institute for Medical Research and Occupational Health, Ksaverska cesta 2, 10000 Zagreb, Croatia; 3 Faculty of Science, University of Sarajevo, Zmaja od Bosne 33-35, 71000 Sarajevo, Bosnia and Herzegovina; sgutic@pmf.unsa.ba; 4 Department of Genetic Toxicology and Cancer Biology, National Institute of Biology, Večna pot 121, 1000 Ljubljana, Slovenia; bojana.zegura@nib.si

**Keywords:** GQDs, FTIR, DNA damage, cell viability, micronuclei

## Abstract

Commercially available graphene quantum dots (GQDs) are promising nanomaterials for applications in research and preclinical diagnostics, drug delivery, and bioimaging. Their bioactivity is highly dependent on dose, route of exposure, duration, cell type, uptake mechanisms, tissue and cellular distribution, and physicochemical properties. This study aimed to evaluate genotoxic, cytotoxic, and cytostatic endpoints of blue- (B-GQDs) and green-emitting (G-GQDs) GQDs in human blood and salivary leukocytes. GQDs were tested at concentrations ranging from 2.5 to 100 µg/mL using distinct treatment periods. Fourier transform infrared spectroscopy (FTIR), trypan blue exclusion, comet, and cytokinesis-block micronucleus cytome (CBMN cyt) assays were performed. FTIR analysis revealed that G-GQDs, unlike B-GQDs, exhibit an absorption band typically associated with amine functional groups, which may contribute to their pronounced genotoxic effects. Peripheral blood mononuclear cells and salivary leukocytes showed higher sensitivity to G-GQDs compared to whole blood samples. Although no cytotoxic effects were observed, both GQDs induced significant DNA damage, with G-GQDs demonstrating greater genotoxic potential. These findings demonstrate that GQDs can induce DNA damage in the absence of detectable cytotoxic effects under the conditions tested, highlighting the importance of considering both physicochemical properties and cellular models in the safety assessment of nanomaterials.

## 1. Introduction

Graphene quantum dots (GQDs) are zero-dimensional carbon-based nanomaterials with characteristic dimensions below 10 nm. They predominantly consist of sp2-hybridised, crystalline carbon structures whose photoluminescence arises from quantum confinement and edge effects. Their structure, together with abundant surface functional groups, provides a high surface area, excellent aqueous dispersibility, and diverse chemical reactivity. These physicochemical properties make GQDs highly attractive for biomedical applications. In particular, their intrinsic photoluminescence, favourable biocompatibility, and low toxicity [1,2] have enabled their widespread use in the biomedical field [3,4,5].

Several physicochemical properties, including particle size, surface chemistry and charge, solubility, and the nature and modification of surface functional groups, influence the biological behaviour of GQDs. These characteristics strongly influence their interactions with biological systems and, consequently, their toxicological profiles. The primary mechanisms underlying GQD-induced toxicity include the generation of reactive oxygen species (ROS) and the induction of oxidative stress, which can lead to DNA damage [6,7], inflammatory responses, apoptosis, and autophagy [8], as well as disturbed cellular homeostasis and mitochondrial/energy-metabolism in vitro [9]. In vivo, exposure to GQDs has been associated with developmental alterations [7], as well as reproductive, behavioural and neurotoxic effects [10]. Despite these findings, data on the cytotoxicity of GQDs remain inconsistent. While several studies have shown low cytotoxicity [11,12,13], others have demonstrated cytotoxic effects at concentrations exceeding 100 µg/mL [14]. Moreover, the genotoxic potential of GQDs has also been reported [15].

A reliable evaluation of the cytotoxic and genotoxic effects of nanoparticles requires the use of appropriate cell models that reflect primary exposure pathways and target organs. Inhalation represents a major route of human exposure and the principal pathway for nanoparticle entry into the body [16,17]. Peripheral blood mononuclear cells (PBMCs) are a well-established and biologically relevant in vitro model for assessing cytotoxic and genotoxic effects [18]. Although obtained from peripheral blood through an inherently invasive procedure, PBMCs are widely used in toxicological and biomonitoring studies as they reflect systemic human exposure [19]. These cells, composed primarily of lymphocytes and monocytes, are highly sensitive to toxic agents [20] and retain functional DNA repair capacity and cell-cycle regulation [21], making them suitable for detecting primary DNA damage and early molecular toxic effects [22]. In parallel, human salivary leukocytes represent a promising, non-invasive model for evaluating genotoxic effects associated with recent inhalational and oral exposures to environmental contaminants. Because of their direct contact with exposed substances, they enable the detection of early, localised DNA damage and are suitable for in vitro genotoxicity assessment, particularly with the comet assay [23].

Given the growing body of evidence supporting the biomedical relevance of graphene quantum dots (GQDs), commercially available GQDs are expected to play an increasingly important role in biomedical research and clinical applications, owing to their superior reproducibility, tunable functionalization, and potential advantages in safety, clearance, and regulatory compliance. The biomedical utility of graphene quantum dots is largely driven by their emission wavelengths, most notably represented by blue (B-GQDs) and green (G-GQDs) emitters [24]. B-GQDs are associated with intrinsic emissive states arising from nanoscale sp^2^ carbon domains, where quantum confinement and edge effects contribute to their photoluminescent properties [25], making them suitable for high-contrast in vitro imaging [26,27] and FRET-based biosensing (Förster or fluorescence resonance energy transfer) [28]. In contrast, the larger, highly oxidised structure of G-GQDs shifts emission toward longer wavelengths, favouring in vivo tracking, environmental chemosensing, and stimuli-triggered drug delivery [29,30,31].

Although both GQDs have a similar nominal particle size (<5 nm, according to the manufacturer), they differ in their photoluminescence properties, with emission maxima at 445 nm (B-GQDs) and 525 nm (G-GQDs). These differences are likely associated with variations in their surface chemistry and functional groups. Our previous physicochemical characterisation showed that G-GQDs exhibit a higher negative zeta potential and better colloidal stability than B-GQDs. At the same time, FTIR analysis (Fourier Transform Infrared spectroscopy) revealed the presence of amine-related functional groups in G-GQDs that were not detected in B-GQDs [32]. Such differences may influence their interactions with biological systems and, consequently, their toxicological profiles.

Since these commercially available GQDs are increasingly used in biomedical research and may be considered for future clinical applications, it is important to assess whether differences in their physicochemical properties lead to distinct biological and genotoxic effects. Accordingly, the present study aimed to evaluate and compare the genotoxic effects of two commercially available GQDs on human peripheral blood cells and salivary leukocytes in vitro.

## 2. Materials and Methods

### 2.1. Chemicals

PB-MAX Karyotyping Medium was obtained from Gibco™, Thermo Fisher Scientific (Waltham, MA, USA). RPMI 1640 medium (Roswell Park Memorial Institute medium), Histopaque^®^-1077, normal melting point (NMP) and low melting point (LMP) agaroses, phosphate-buffered saline (PBS), trypan blue, absolute ethanol, sodium hydroxide (NaOH), sodium chloride (NaCl), mitomycin C, cytochalasin B, dimethyl sulfoxide (DMSO), DAPI (4′,6-diamidino-2-phenylindole), hydrogen peroxide solution (H_2_O_2_), glacial acetic acid as well as B-GQDs and G-GQDs were purchased from Sigma (St. Louis, MO, USA). Disodium hydrogen phosphate (Na_2_HPO_4_) and disodium EDTA (Na_2_EDTA) were obtained from Kemika (Zagreb, Croatia), while potassium dihydrogen phosphate (KH_2_PO_4_) was purchased from Centrohem (Stara Pazova, Serbia). Tris-HCl was obtained from HiMedia Laboratories (Mumbai, Maharashtra, India), and Triton X-100 from PlosOne Pharmacia Biotech AB (Uppsala, Sweden). Potassium chloride (KCl) was obtained from Carlo Erba (Milan, Italy).

### 2.2. GQD Preparation and Treatments

A detailed physicochemical characterisation of the tested GQDs, including transmission electron microscopy (TEM) (Jeol 2100, JEOL, Tokyo, Japan), zeta potential, and hydrodynamic radius (Litesizer DIF 500, Anton Paar GmbH, Gratz, Austria), has been previously described [32]. The tested materials were commercially available blue-emitting (B-GQDs) and green-emitting (G-GQDs) graphene quantum dots (Sigma-Aldrich, St. Louis, MO, USA). According to the manufacturer’s specifications, both materials have a nominal particle diameter below 5 nm but differ in their fluorescence properties, exhibiting emission maxima at 445 nm and 525 nm for B-GQDs and G-GQDs, respectively. Aggregation/agglomeration behaviour under cell culture conditions was not assessed in the present study. However, detailed physicochemical characterisation of the same materials, including transmission electron microscopy (TEM), zeta potential, and hydrodynamic radius measurements, has been reported previously [32]. Briefly, G-GQDs showed a more negative zeta potential in water (−51.6 mV) than B-GQDs (−18.1 mV), indicating greater colloidal stability. Hydrodynamic radius measurements revealed aggregation in aqueous suspensions, particularly for B-GQDs, whereas markedly smaller particle sizes were observed in cell culture medium (approximately 25–27 nm), suggesting improved dispersion under exposure conditions [32]. Stock solutions of B-GQDs and G-GQDs (1 mg/mL) were prepared directly in the corresponding cell culture media and mixed thoroughly prior to use. Final concentrations of 2.5, 5, 10, and 100 µg/mL were achieved by directly adding the required volumes of the respective stock solutions to the cell cultures. Untreated cell cultures served as the negative control (NC), while assay-specific treatments served as positive controls (PCs).

### 2.3. Physicochemical Characterisation

A detailed physicochemical characterisation of both tested GQDs has been reported previously [32]. Transmission electron microscopy (TEM) analysis confirmed their nanoscale size and predominantly spherical morphology. In aqueous medium, G-GQDs exhibited a higher negative zeta potential (−51.6 mV) compared to B-GQDs (−18.1 mV), indicating better colloidal stability. The hydrodynamic radius was large for both samples in water (G-GQDs: 1209.4 nm, B-GQDs: 8086.1 nm) due to aggregation, consistent with TEM observations. To complement these previously reported data and provide additional information on the surface chemistry of the tested GQDs, Fourier transform infrared spectroscopy (FTIR) analysis was performed in the present study. FTIR spectra were recorded in attenuated total reflectance (ATR) mode using a PerkinElmer UATR Two spectrometer. Powder samples were applied directly to the ATR crystal at room temperature. The spectra were collected over the range of 400–4000 cm^−1^ with a spectral resolution of 1 cm^−1^. Aggregation/agglomeration behaviour under cell culture conditions was not assessed in the present study.

### 2.4. Ethical Approval and Informed Consent

The study and all experimental procedures were approved by the Ethics Committee of the University of Sarajevo—Institute for Genetic Engineering and Biotechnology (Approval No. 204/25, dated 13 May 2025, and 516/25, dated 21 November 2025). Written informed consent was obtained from all participants before their inclusion in the study.

### 2.5. PBMCs Isolation

Blood samples were obtained from three healthy female volunteers, each serving as an independent experimental replicate. Peripheral blood was collected into heparinised vacuum tubes. PBMCs were isolated by density gradient centrifugation as described by Pires et al. [33], using Histopaque-1077 according to the manufacturer’s instructions. Briefly, whole blood, without previous dilution, was layered onto Histopaque-1077 at a 1:1 ratio and centrifuged at 400× *g* for 30 min at room temperature with slow acceleration and no brake. Following centrifugation, the mononuclear cell layer was carefully aspirated and transferred into sterile centrifuge tubes. The collected cells were washed three times with PBS, resuspended, and cultured in complete medium containing mitosis stimulator phytohemagglutinin, L-glutamine, and antibiotics (PB-MAX™, Karyotyping Medium) at a final concentration of 1 × 10^6^ cells/mL. Cell cultures were incubated at 37 °C in a humidified 5% CO_2_ atmosphere for 48 h in total and subsequently used for trypan blue exclusion and comet assays. Cells were treated after 24 h of incubation for the trypan blue assay and after 45 h of incubation for the comet assay.

### 2.6. Trypan Blue Exclusion Assay on PBMCs

After 24 h of incubation, the PBMC cultures were treated with GQDs for an additional 24 h (37 °C, 5% CO_2_). A 5% DMSO solution served as the positive control. Following treatment, the cell cultures were centrifuged at 117× *g* for 5 min at room temperature. The trypan blue exclusion assay was performed according to Strober [34], using a LUNA-II Automated Cell Counter (Logos Biosystems, Anyang, South Korea) to assess cell count and viability. PBMCs were selected for this assay due to their greater sensitivity than the other cellular model used, namely, salivary leukocytes. The trypan blue exclusion assay was used to ensure that subsequent genotoxicity analyses were performed at non-cytotoxic concentrations of the tested GQDs, in accordance with OECD recommendations for in vitro genotoxicity testing [35,36] and for evaluating the biological effects of manufactured nanomaterials [37]. As this assay directly evaluates cell membrane integrity and allows reliable discrimination between viable and non-viable cells [34], it was selected over the MTT assay, which primarily reflects metabolic activity rather than cell death.

### 2.7. Comet Assay on PBMCs

To determine the level of primary DNA damage, the alkaline comet assay was carried out as recommended by the MIRCA protocol [38] and Cetkovic et al. [39], with minor modifications, including a shortened hydrogen peroxide treatment for the positive control and a shorter lysis duration. During the final 3 h of the 48 h incubation, cells were treated with GQDs (2.5, 5, 10, and 100 μg/mL). After treatment, cells were centrifuged at 117× *g* for 5 min. Cell suspension (120 μL) was mixed with 180 μL of 0.7% LMP agarose and layered onto slides coated with 1% NMP agarose. Positive control (PC) slides were prepared by briefly immersing untreated slides in 70 µM H_2_O_2_ at 4 °C for 30 s [40]. Subsequently, slides were placed in freshly prepared cold lysis solution for 1 h at 4 °C. After cell lysis, the slides were placed in freshly prepared cold electrophoresis solution for 20 min to allow DNA unwinding, followed by electrophoresis at 1 V/cm for an additional 20 min, both at 4 °C. After electrophoresis, slides were rinsed with PBS, fixed sequentially in 70% and absolute ethanol (5 and 15 min, respectively), then rehydrated and stained with DAPI (1 µg/mL) prior to analysis using an epifluorescent microscope (Olympus BX51, Tokyo, Japan). Three independent experiments were performed, each time analysing 200 randomly selected nuclei per condition. DNA damage was quantified using Comet Assay IV software version 4.3 (Instem plc, Stone, UK) by measuring tail intensity (%TI).

### 2.8. Salivary Leukocytes Isolation

Saliva samples were obtained from six healthy, non-smoking female volunteers and processed as described by Cetkovic Pecar et al. [41]. Donors were instructed to refrain from eating or drinking anything except water for at least 1 h prior to sampling. Saliva samples were collected from each participant by two consecutive mouth rinses, each lasting 1 min, using 20 mL of sterile 0.9% NaCl solution. The two rinses were pooled into sterile 50 mL centrifuge tubes and centrifuged at 1100× *g* for 15 min at 4 °C without a brake. The supernatant was discarded, and the cell pellets were washed with 10 mL PBS under the same conditions. Leukocyte isolation was performed as described by Valdiglesias et al. [23], with minor modifications. Samples were centrifuged at 1100× *g* for 15 min at 4 °C, and the pellets were resuspended in 6 mL of RPMI 1640 medium, layered over 3 mL of Histopaque^®^-1077, and centrifuged at 400× *g* for 30 min at 4 °C without a brake.

The leukocyte-containing interface was collected into 1.5 mL tubes and centrifuged at 1100× *g* for 25 min at 4 °C without a brake. The supernatant was discarded, and the pellet was washed with 1 mL PBS and centrifuged again for 15 min under the same conditions. After removal of the supernatant (1 mL), the pellet was finally resuspended in 500 μL RPMI 1640 medium. Cells from all donors were pooled, counted using LUNA-II™ Automated Cell Counter (Logos Biosystems, Anyang, South Korea), and incubated in sterile RPMI 1640 medium at 1 × 10^6^ cells/mL at 37 °C in a humidified 5% CO_2_ atmosphere. Cells were immediately treated with GQDs at final concentrations of 2.5, 5, 10, and 100 μg/mL for 3 h.

### 2.9. Comet Assay on Salivary Leukocytes

Salivary leukocytes were centrifuged at 117× *g* for 5 min after 3 h of treatment. The comet assay was performed following the same protocol described for PBMCs. DNA damage was evaluated using the same parameter (tail intensity, %TI) as for PBMCs, and experiments were performed in triplicate.

### 2.10. CBMN Cyt Assay

The genotoxic, cytotoxic, and cytostatic potential of GQDs was assessed in whole blood cultures using the cytokinesis-block micronucleus cytome (CBMN cyt) assay. Cultures were established in 15 mL sterile tubes containing 5 mL PB-MAX Karyotyping Medium and 400 μL peripheral blood. After 24 h, cells were treated with the indicated concentrations of GQDs, with mitomycin C (0.5 µg/mL) as a positive control. Cytokinesis was blocked by adding cytochalasin B at the 45th hour to a final concentration of 4.5 μg/mL. Exposing cells to graphene-based materials before adding cytochalasin B aligns with OECD guidelines to ensure nanomaterial uptake before endocytosis is blocked [42]. The assay was performed without S9 metabolic activation as nanomaterial-induced genotoxicity is generally mediated either directly by interaction with DNA, or indirectly through the generation of reactive oxygen species, and most nanomaterials do not require metabolic activation to induce a genotoxic response [43,44]. Cultures were harvested after 72 h of incubation at 37 °C, following standard procedures, including hypotonic treatment, fixation, slide preparation, and staining with 5% Giemsa [45]. Microscopic analysis was performed using a KERN OBL 137 light microscope (KERN & SOHN GmbH, Ballingen, Germany) at 400× magnification. For each treatment and control, a total of 4000 binucleated cells were analysed, divided into four replicates of 1000 cells each. Genotoxicity was assessed by scoring micronuclei (MNi), nucleoplasmic bridges (NPB), and nuclear buds (NB) [46,47]. At the same time, cytotoxic and cytostatic effects were evaluated using the nuclear division index (*NDI*) and nuclear division cytotoxicity index (*NDCI*) [48]. The cytokinesis-block proliferation index (*CBPI*) [35,49] and the Replication Index (*RI*) [35,50], which reflect treatment-related inhibition of cell proliferation, were calculated as well.

Formulas are provided below:

(1) $$NDI=\frac{\left(M1\right)+2\times \left(M2\right)+3\times \left(M3\right)+4\times (M4)}{N}$$

(2) $$NDCI=\frac{Ap+Nec+\left(M1\right)+2\times \left(M2\right)+3\times \left(M3\right)+4\times (M4)}{N1}$$

(3) $$CBPI=\frac{\left(M1\right)+2\times \left(M2\right)+3\times (M3+M4)}{N}$$

(4) $$RI=\frac{\frac{{\left(M2\right)}_{T}+2\times {\left(M3+M4\right)}_{T}}{{\left(N\right)}_{T}}}{\frac{{\left(M2\right)}_{C}+2\times {\left(M3+M4\right)}_{C}}{{\left(N\right)}_{C}}}\times 100$$

*M*1 to *M*4—numbers of cells with one to four nuclei; *N*—total number of viable cells scored; *N*1—total number of viable and nonviable cells scored; *Ap*—number of apoptotic cells; *Nec*—number of necrotic cells; *T*—treated cultures; *C*—control cultures.

### 2.11. Statistical Analysis

Comet assay results were log-transformed to normalise data distribution and equalise variances. Statistical analyses were performed using GraphPad Prism version 10.6.1 (GraphPad Software, Boston, MA, USA). Data obtained from the trypan blue, comet, and CBMN cyt assays, including endpoints related to cytotoxic, genotoxic, and cytostatic potential, were tested for significant differences between tested concentrations and the corresponding controls using one-way analysis of variance (ANOVA), followed by Tukey’s multiple comparisons test. A *p* < 0.05 was considered statistically significant.

## 3. Results

### 3.1. Fourier Transform Infrared Spectroscopy (FTIR)

**Figure 1:**
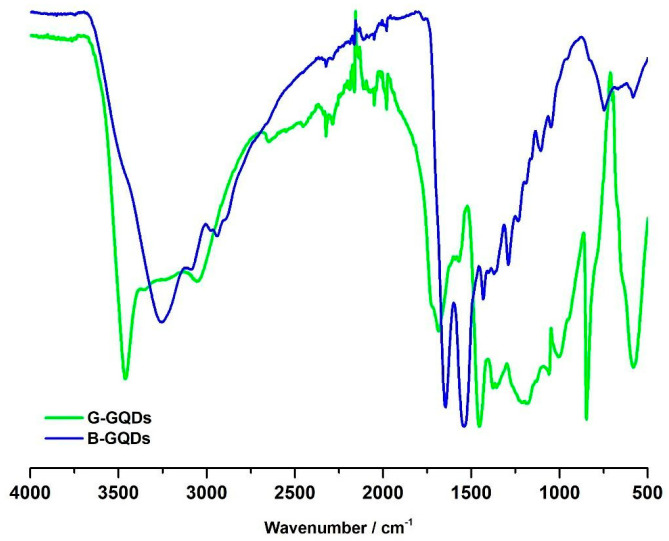
FTIR spectra of graphene quantum dot samples.

The FTIR spectra of the investigated materials indicate the presence of various functional groups. The most pronounced difference between the samples was observed in the 3000–3700 cm^−1^ region (Figure 1), which is characteristic of O–H stretching vibrations associated with molecules of adsorbed water in graphene-based materials, as well as –NH_2_ groups attached to the aromatic structure. G-GQDs exhibit an absorption band at approximately 3460 cm^−1^, which can be attributed to the presence of amine groups bonded to the aromatic framework. In contrast, B-GQDs did not show absorption bands in this region that are typically associated with amine functional groups.

### 3.2. Results of Trypan Blue Exclusion Assay on PBMCs

**Figure 2:**
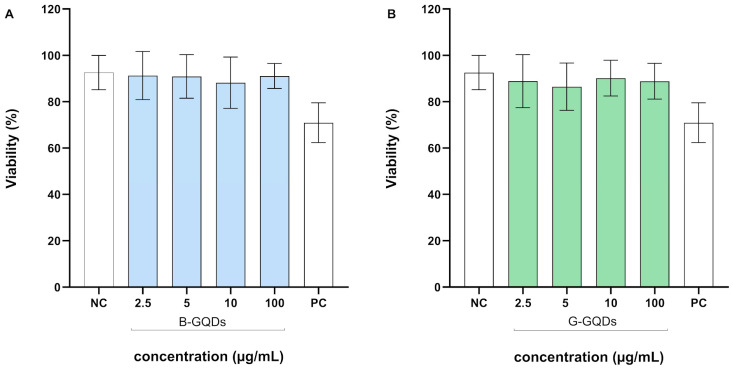
Cell viability in PBMCs after 24 h treatment with blue- (B-GQDs) (**A**) and green-emitting GQDs (G-GQDs) (**B**), as assessed by the trypan blue exclusion assay. NC is the negative control (cell growth media); PC is the positive control (5% DMSO). Data are presented as mean ± standard deviation (SD) from three independent experiments.

Cell viability of PBMCs following exposure to B-GQDs (Figure 2A) and G-GQDs (Figure 2B) was assessed at concentrations ranging from 2.5 to 100 µg/mL. For both GQDs, cell viability remained high across all tested concentrations, ranging from approximately 86% to 91%, with no clear dose-dependent decrease compared to the negative control (93%).

### 3.3. Results of Comet Assay on PBMCs

**Figure 3:**
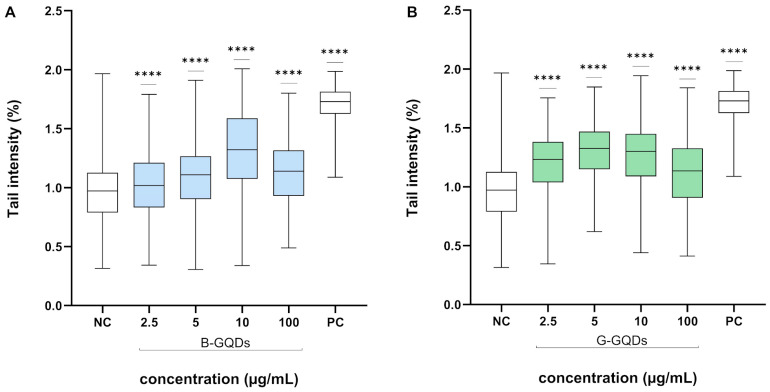
Tail intensity (%TI) in PBMCs following exposure to blue- (B-GQDs) (**A**) and green-emitting GQDs (G-GQDs) (**B**) evaluated by the comet assay. Data are presented as mean ± standard deviation (SD) from three independent experiments of log-transformed data. NC is the negative control (cell growth media); PC is the positive control (70 µM H_2_O_2_). Asterisks indicate statistically significant differences between the negative control and exposed cells, as determined by ANOVA followed by Tukey’s multiple comparisons test (**** *p* < 0.0001).

DNA damage in PBMCs exposed to B-GQDs (Figure 3A) and G-GQDs (Figure 3B) was evaluated by measuring tail intensity. Both materials induced a statistically significant increase in TI across all tested concentrations compared with the negative control (**** *p* < 0.0001). Across comparable concentration levels, at the lower tested concentrations (2.5 and 5 µg/mL), G-GQDs induced significantly higher TI than B-GQDs (**** *p* < 0.0001). Although statistically significant increases in %TI were observed following GQD exposure, the absolute levels of DNA damage remained relatively low and were markedly lower than those induced by the positive control (Figure 3A,B). Therefore, the observed DNA damage should be interpreted as a modest but measurable genotoxic response under the applied experimental conditions.

### 3.4. Results of Comet Assay on Salivary Leukocytes

**Figure 4:**
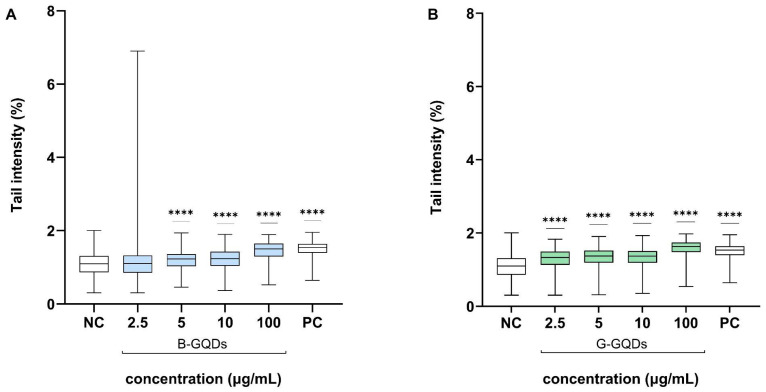
Tail intensity (%TI) in salivary leukocytes following exposure to blue- B-GQDs (**A**) and green-emitting GQDs (G-GQDs) (**B**) evaluated by the comet assay. Data are presented as mean ± standard deviation (SD) from three independent experiments of log-transformed data. NC is the negative control (cell growth media); PC is the positive control (70 µM H_2_O_2_). Asterisks ndicate statistically significant differences between the negative control and exposed cells, as determined by ANOVA followed by Tukey’s multiple comparisons test (**** *p* < 0.0001).

The comet assay performed on salivary leukocytes showed concentration-dependent changes in the primary DNA damage parameter (%TI) following exposure to B-GQDs and G-GQDs. For B-GQDs, significant differences relative to the negative control were detected at all tested concentrations except the lowest (Figure 4A). The relatively broad distribution observed at 2.5 µg/mL most likely reflects biological variability inherent to primary human salivary leukocytes and the low magnitude of the response at this concentration. Since no clear concentration-dependent increase in variability was observed at higher concentrations, we do not believe that this finding reflects a systematic treatment-related effect. In contrast, for G-GQDs, a significant increase in TI was observed at all tested concentrations (Figure 4B) (**** *p* < 0.0001). When tested at the same concentration levels, G-GQDs showed significantly higher TI values compared to B-GQDs (**** *p* < 0.0001).

### 3.5. Results of CBMN Cyt Assay

The results of the genotoxicity assessment of GQDs in human lymphocyte cultures, as determined by the CBMN cyt assay, are presented in Table 1. Both GQDs induced concentration-dependent changes in CBMN cyt biomarkers. For G-GQDs, a significant increase in the frequency of micronuclei was observed at 100 µg/mL (** *p* < 0.005) compared with the negative control, corresponding to an approximately 2.8-fold increase. In the case of B-GQDs, micronucleus (MN) frequency was significantly increased at 10 µg/mL (* *p* < 0.05), with a highly significant increase in total MNi at 100 µg/mL (*** *p* < 0.0005), corresponding to an approximately 3.6-fold increase compared to the negative control. Notably, only the highest concentration of B-GQDs (100 µg/mL) resulted in a ≥2-fold increase in MNi, meeting the commonly accepted criterion for a biologically relevant positive response. For both GQD types, slight increases in nuclear buds (NBs) and nucleoplasmic bridges (NPBs) were observed at higher concentrations; however, these changes did not reach statistical significance. *NDI*, *NDCI*, *CBPI* and *RI* values are presented in Table 2. *CBPI* is similar to *NDI*, but it estimates the average number of cell divisions completed by the cell population; therefore, trinucleated and tetranucleated cells are grouped within the same category [49]. Calculated *NDI* and *NDCI* remained largely stable across all concentrations of both GQDs, with no significant deviations from the negative control, indicating that overall cell proliferation and cytotoxicity parameters were not meaningfully affected under the experimental conditions. However, *RI* values following treatment with B-GQDs at 2.5 µg/mL were significantly lower than those observed after treatment with both B-GQDs and G-GQDs at higher concentrations (5, 10, and 100 µg/mL). No significant difference was observed between B-GQDs at 2.5 µg/mL and the positive control.

## 4. Discussion

Graphene quantum dots (GQDs), highly crystalline graphene fragments exhibiting quantum confinement effects due to their sp^2^-hybridized carbon structure, have been widely recognised for their biomedical and commercial potential. This is largely attributed to their small size, high dispersibility, efficient cellular uptake, and generally favourable biocompatibility profile, including relatively low cytotoxicity [12,51]. In addition, GQDs exhibit strong solubility and a reduced tendency to aggregate compared to larger graphene-based nanomaterials and other quantum dots [52,53], further supporting their applicability in biological systems.

In this study, the biological responses of human peripheral blood mononuclear cells (PBMCs) and salivary leukocytes to two commercially available GQDs were assessed in vitro, including genotoxic, cytotoxic, and cytostatic endpoints. The combined use of these two cell models enabled a more comprehensive evaluation of genotoxic potential: PBMCs provide insight into systemic effects, whereas salivary leukocytes offer a non-invasive, ethically advantageous model for assessing early exposure-related genotoxic damage, particularly relevant to inhalation and oral exposure routes. However, an important methodological distinction between the models should be considered. PBMCs were cultured in PB-MAX medium, which stimulates lymphocyte proliferation, whereas salivary leukocytes were analysed in a non-proliferating state. Since proliferative status and cellular activation may influence sensitivity to DNA-damaging agents through differences in nanoparticle uptake, metabolism, cell-cycle progression, and DNA repair capacity, direct quantitative comparisons between PBMCs and salivary leukocytes should be interpreted with caution [54,55]. Importantly, although GQDs are often considered low-toxicity nanomaterials, their biological effects depend strongly on their physicochemical properties, including size, surface chemistry, and functionalization. Therefore, the evaluation of commercially available GQDs is essential, as these materials are increasingly used in research and potential clinical applications, yet their safety profiles remain insufficiently characterised.

Previous studies have demonstrated that the surface functionalization of GQDs plays a crucial role in determining their biological activity [56]. In the present study, no significant differences in cell viability were observed between GQD-treated and control PBMCs across all tested concentrations, indicating that the GQDs did not affect cell viability under these conditions. These findings are consistent with those reported by Yuan et al. [13], which describe the low cytotoxicity of GQDs (0–200 µg/mL) in human lung adenocarcinoma epithelial (A549) cells.

In contrast, our previous research investigating the cytotoxic effects of B-GQDs and G-GQDs in human hepatocellular carcinoma (HepG2) 3D spheroids revealed a different response pattern. B-GQDs exhibited significant cytotoxicity only at 250 µg/mL, while G-GQDs demonstrated more pronounced cytotoxic effects at both 100 µg/mL and 250 µg/mL [32]. Based on these findings, and particularly the cytotoxic effect observed at 250 µg/mL, the present study was limited to concentrations of up to 100 µg/mL.

As mentioned, the biological effects of GQDs are influenced by their physicochemical characteristics and exposure conditions. Notably, variations in synthesis routes, surface chemistry, and GQD modifications can significantly affect cellular uptake, intracellular distribution [13], antioxidant properties [57], overall bioactivity [12], and toxicity profiles [11,14].

For example, hydroxyl-functionalized GQDs (–OH) have been shown to induce cytotoxic effects in A549 cells at concentrations exceeding 100 µg/mL, as assessed by the WST-1 assay [14], suggesting that both surface chemistry and dose are critical determinants of toxicity. These factors should therefore be carefully considered when interpreting and comparing toxicological outcomes across different studies.

The trypan blue exclusion assay was selected over the MTT assay, which primarily reflects metabolic activity rather than cell death. In addition, potential interference by nanomaterials with tetrazolium-based assays (e.g., MTT, XTT, WST-1) has been reported previously [58], further supporting the use of trypan blue in this study. The different exposure durations were applied depending on the toxicological endpoint assessed. The trypan blue assay was conducted after 24 h of treatment, while genotoxicity assessment included three and 48 h treatment periods. These time points reflect the distinct toxicological endpoints with different temporal requirements. A short-term treatment (3 h) for the comet assay was applied to detect early primary DNA damage, including transient DNA strand breaks and alkali-labile sites, before extensive DNA repair or secondary cellular responses occur in PBMCs [21] and salivary leukocytes [23] of 24 h for the trypan blue assay to capture delayed membrane damage and 48 h for the cytokinesis-block micronucleus assay in blood leukocytes to allow mitogen-stimulated lymphocytes to complete cell division and express chromosomal damage as micronuclei, a post-mitotic endpoint. Additionally, Babonaite et al. [59] reported that nanoparticle-induced DNA damage was more pronounced after 24 h than after 3 h, as DNA damage may increase with exposure time due to delayed cellular uptake and intracellular interactions. Exposing cells to graphene-based materials before cytochalasin B treatment prevents false-negative results by allowing internalisation and interaction with target organelles during the cell cycle. Cytochalasin B, often used to inhibit cytokinesis, may also inhibit endocytosis, leading to false-negative outcomes with nanomaterials. Possible interference from nanomaterial deposits during micronuclei scoring at high test concentrations should also be considered [42]. Genotoxicity assessment using the comet assay revealed that G-GQDs exhibited higher genotoxic activity than B-GQDs, inducing significant DNA strand breaks in PBMCs and salivary leukocytes at all tested concentrations (2.5, 5, 10, and 100 µg/mL) compared to the negative control. In our previous study evaluating the genotoxicity of the same materials in a 3D HepG2 model, both B-GQDs and G-GQDs induced a pronounced increase in tail intensity at 25 µg/mL [32].

*NDI* represents the average number of nuclei per cell, while *CBPI* provides an estimate of the average number of cell cycles completed by the cell population, which may be more biologically meaningful in cytokinesis-blocked cells [49]. In parallel, *NDCI* may be more biologically comprehensive because it also includes apoptotic and necrotic cells. Accordingly, reporting all four indexes (*NDI*, *NDCI*, *CBPI*, and *RI*) allows for more inclusive comparison with the existing literature in this research area. Analysis of CBMN cyt assay parameters showed that *NDI* values remained within a narrow interval across all treatments, indicating that cell proliferation was not adversely affected. Similarly, *NDCI* values showed no consistent dose-dependent trend, suggesting no marked cytotoxic effects under the experimental conditions. Overall, these findings indicate that, despite the observed increases in MN frequency, GQDs did not significantly impair cell division kinetics. Significantly lower value of *RI*, observed after treatment with B-GQDs at 2.5 µg/mL, may be related to concentration-dependent differences in nanoparticle dispersion and cellular availability. At lower concentrations, nanoparticles may remain better dispersed and more available for cell interaction.

Nevertheless, at 100 µg/mL, both G-GQDs and B-GQDs significantly increased micronucleus frequency in human lymphocytes, confirming a strong genotoxic effect at the highest tested concentration (approximately 2.8- and 3.6-fold increases over the negative control for G-GQDs and B-GQDs, respectively). These results align with previous observations of hydroxyl-functionalized GQDs inducing MN formation in human epithelial HET-1A cells at a concentration of 100 µg/mL [6].

The observed differences in genotoxicity between G-GQDs and B-GQDs can be explained by their distinct physicochemical characteristics. Morphological analysis of the tested GQDs [32] showed that B-GQDs had an average size of approximately 20 nm. In contrast, G-GQDs displayed a bimodal size distribution, with populations below 10 nm and around 20 nm. The presence of smaller particles in the G-GQD sample may contribute to increased surface reactivity and enhanced biological interactions, potentially explaining their higher genotoxic potential. Zeta potential measurements further indicated that G-GQDs had a higher absolute negative charge in water (−51.6 mV vs. −18.1 mV for B-GQDs), consistent with better colloidal stability. In cell culture medium, both types of GQDs exhibited reduced zeta potential and hydrodynamic radius, likely due to protein corona formation and ionic screening effects, which may stabilise the nanoparticles and reduce large-scale aggregation [32].

In addition, the differences observed in the FTIR spectra, particularly the presence of an –NH_2_ group bound to the aromatic ring in G-GQDs, may explain the genotoxic effect observed compared to B-GQDs. The findings of Yuan et al. [13] also revealed a significant decrease in cell proliferation in A549 cells treated with NH_2_-GQDs at concentrations of 100 and 200 µg/mL compared with the negative control.

The observed differences in genotoxicity may also be related to the photoluminescence properties of the tested GQDs. According to the manufacturer’s specifications (Sigma, St. Louis, MO, USA), B-GQDs exhibit excitation/emission maxima at 350/445 ± 10 nm, whereas G-GQDs emit at 485/525 nm. Blue-emitting GQDs exhibit aggregation-induced concentration quenching at higher concentrations, suggesting a tendency to form larger aggregates that may reduce their cellular uptake and reactivity. In contrast, green-emitting GQDs show fluorescence enhancement at higher concentrations, indicating improved colloidal stability and a higher fraction of smaller, potentially more reactive particles [60]. These properties may influence cellular interactions, uptake, and intracellular distribution. Although cellular uptake of graphene quantum dots was not directly assessed in the present study, previous reports have shown that graphene quantum dots enter cells via endocytosis and accumulate in the nucleus, where they can induce DNA strand breaks and micronucleus formation during mitosis [6,61], which may explain the genotoxic effects observed in the present study.

Both tested GQDs induced significantly higher TI in salivary leukocytes compared to PBMCs at concentrations of 10 and 100 µg/mL (*p* < 0.005). These findings suggest that salivary leukocytes may be more susceptible to GQD-induced genotoxic effects than PBMCs. The observed differences in genotoxic responses between PBMCs and salivary leukocytes, relative to whole-blood samples, may be explained by differences in cellular composition and nanoparticle uptake. Whole blood preserves the complete cellular and plasma environment, including erythrocytes, platelets, circulating biomolecules, and antioxidant components, which may influence nanoparticle bioavailability, distribution and the overall cellular response to oxidative stress. In contrast, PBMCs contain metabolically active lymphocytes and monocytes that can efficiently internalise nanoparticles, potentially increasing intracellular accumulation and oxidative stress [6]. Additionally, salivary leukocytes, which consist predominantly of neutrophils, may exhibit distinct responses to nanoparticle exposure [23]. Variations between genotoxicity endpoints may reflect differences in the types of DNA damage assessed and the corresponding cellular responses. The comet assay primarily detects early DNA damage that is potentially repairable [62], whereas the CBMN-cyt assay detects irreversible chromosomal damage that becomes evident after cell division [63]. Therefore, variations in genotoxic responses among whole blood, PBMCs, and salivary leukocytes may also be influenced by differences in DNA repair capacity and proliferative activity of these cell types, ultimately affecting the progression from initial DNA lesions to fixed chromosomal damage.

Accordingly, occupational and biomedical exposure to GQDs, especially G-GQDs, should be carefully considered. Based on our findings, salivary leukocytes showed sensitivity even at low concentrations of GQDs, suggesting their potential as a relevant non-invasive model for assessing low-level exposure, likely reflecting realistic human scenarios. These findings demonstrate that GQDs can exert genotoxic effects independently of cytotoxicity and highlight the importance of employing multiple complementary endpoints in nanomaterial safety assessment.

## 5. Conclusions

In conclusion, this study demonstrates that commercially available blue- (B-GQDs) and green-emitting (G-GQDs) graphene quantum dots induce significant genotoxic effects in human primary cell models in the complete absence of detectable cytotoxicity. Green-emitting GQDs exhibit a more pronounced genotoxic potential at lower concentrations, likely due to their amine surface functionalization (−NH_2_) and smaller particle fractions. Furthermore, isolated peripheral blood mononuclear cells and salivary leukocytes exhibit heightened sensitivity to GQD-induced primary DNA damage compared with whole-blood environments. Notably, these findings validate human salivary leukocytes as a highly sensitive, non-invasive screening tool capable of capturing low-level, localised nanomaterial exposures. From a toxicological safety perspective, our data highlight a critical risk: quantum dots can cause severe, irreversible chromosomal damage without altering cell membrane integrity or survival kinetics. Relying solely on conventional cell viability assays can therefore mask underlying genotoxicity, leading to false-negative safety profiles during early risk assessments. Consequently, comprehensive safety screening for carbon-based nanomaterials must mandate multi-endpoint testing strategies that combine transient DNA lesion profiling with post-mitotic chromosomal assessments. Moving forward, further mechanistic investigations are required to track the direct nuclear interactions, endocytosis pathways, and long-term consequences of fixed chromosomal damage to safely guide the occupational and biomedical translation of these quantum dots.

## Figures and Tables

**Table 1:** Frequency of CBMN cyt genotoxicity biomarkers (micronuclei (MNi), nuclear buds (NBs), and nucleoplasmic bridges (NPBs)) in human lymphocyte cultures following exposure to B-GQDs and G-GQDs.

Treatments (µg/mL)		Total MNi	NBs	NPBs
B-GQDs	2.5	15.75 ± 5.74	2.5 ± 1.29	2.25 ± 0.96
	5	14 ± 6.98	1.25 ± 1.89	1.25 ± 1.26
	10	26.5 ± 12.12 *	3 ± 3.46	1.5 ± 0.58
	100	35 ± 2.16 ***	5.5 ± 2.08	2.25 ± 0.96
G-GQDs	2.5	17.25 ± 4.03	2.25 ± 2.22	0.75 ± 0.96
	5	14.75 ± 4.65	2 ± 0.82	0.75 ± 0.96
	10	14.75 ± 6.8	3.25 ± 0.96	0.75 ± 0.96
	100	27 ± 4.76 **	3.5 ± 1.00	3.25 ± 1.89
Negative control (NC)		9.75 ± 2.87	2 ± 1.41	0.25 ± 0.5
Positive control (PC)		50 ± 11.83	5.75 ± 2.50	2.25 ± 0.96

Data are presented as mean ± standard deviation (SD) from four independent experiments. The frequency was expressed per 1000 binucleated cells, while a total of 4000 binucleated cells were scored for each tested condition. Asterisks indicate statistically significant differences between the negative control and exposed cells, as determined by ANOVA followed by Tukey’s multiple comparisons test (* *p* < 0.05; ** *p* < 0.005; *** *p* < 0.0005). Mitomycin C (0.5 µg/mL) was used as the positive control.

**Table 2:** Cytostasis and cytotoxicity measures, nuclear division indexes (*NDI*/*NDCI*) and proliferation indexes (*CBPI*/*RI*) in human lymphocyte cultures following exposure to B-GQDs and G-GQDs.

Treatments (µg/mL)		*NDI*	*NDCI*	*CBPI*	*RI*
B-GQDs	2.5	1.417 ± 0.06	1.411 ± 0.06	1.375 ± 0.06	32.232 ± 4.75 *
	5	1.372 ± 0.03	1.366 ± 0.03	1.338 ± 0.02	81.524 ± 17.02
	10	1.369 ± 0.09	1.364 ± 0.1	1.332 ± 0.08	80.252 ± 24.30
	100	1.472 ± 0.01	1.466 ± 0.02	1.416 ± 0.01	100.108 ± 17.28
G-GQDs	2.5	1.345 ± 0.07	1.340 ± 0.07	1.312 ± 0.05	74.180 ± 10.87
	5	1.389 ± 0.06	1.383 ± 0.06	1.354 ± 0.05	85.189 ± 18.77
	10	1.371 ± 0.05	1.364 ± 0.05	1.340 ± 0.05	91.732 ± 14.75
	100	1.498 ± 0.06	1.493 ± 0.06	1.445 ± 0.05	108.218 ± 29.08
Negative control (NC)		1.479 ± 0.08	1.475 ± 0.08	1.425 ± 0.07	-
Positive control (PC)		1.334 ± 0.02	1.329 ± 0.03	1.303 ± 0.03	73.621 ± 18.55

Data are presented as mean ± standard deviation (SD) from four independent experiments, each performed in at least 500 cells. Total of 2000 cells were scored for each treatment. *NDI*: Nuclear Division Index; *NDCI*: Nuclear Division Cytotoxicity Index; *CBPI*: Cytokinesis-Block Proliferation Index; *RI*: Replication Index. Asterisks indicate significantly lower values compared to B-GQD and G-GQD treatments (5, 10, 100 µg/mL), as determined by ANOVA followed by Tukey’s multiple comparisons test (* *p* < 0.001). Mitomycin C (0.5 µg/mL) was used as the positive control.

## Data Availability

The original contributions presented in this study are included in the article. Further inquiries can be directed to the corresponding authors.

## Abbreviations

The following abbreviations are used in this manuscript:

GQDs	Graphene quantum dots
B-GQDs	Blue-emitting graphene quantum dots
G-GQDs	Green-emitting graphene quantum dots
FTIR	Fourier transform infrared spectroscopy
ATR	Attenuated total reflectance
CBMN cyt	Cytokinesis-block micronucleus cytome assay
ROS	Reactive oxygen species
PBMCs	Peripheral blood mononuclear cells
RPMI 1640	Roswell Park Memorial Institute medium
NMP	Normal melting point (agarose)
LMP	Low melting point (agarose)
PBS	Phosphate-buffered saline
NaOH	Sodium hydroxide
NaCl	Sodium chloride
DMSO	Dimethyl sulfoxide
DAPI	4′,6-diamidino-2-phenylindole
Na_2_HPO_4_	Disodium hydrogen phosphate
Na_2_EDTA	Disodium EDTA
KH_2_PO_4_	Potassium dihydrogen phosphate
KCl	Potassium chloride
H_2_O_2_	Hydrogen peroxide
TEM	Transmission electron microscopy
TI	Tail intensity
MNi	Micronuclei
NB/NBs	Nuclear bud(s)
NPB/NPBs	Nucleoplasmic bridge(s)
*NDI*	Nuclear division index
*NDCI*	Nuclear division cytotoxicity index
*CBPI*	Cytokinesis-block proliferation index
*RI*	Replication index
ANOVA	Analysis of variance
